# Real-time near infrared artificial intelligence using scalable non-expert crowdsourcing in colorectal surgery

**DOI:** 10.1038/s41746-024-01095-8

**Published:** 2024-04-22

**Authors:** Garrett Skinner, Tina Chen, Gabriel Jentis, Yao Liu, Christopher McCulloh, Alan Harzman, Emily Huang, Matthew Kalady, Peter Kim

**Affiliations:** 1 Jacobs School of Medicine and Biomedical Sciences, University at Buffalo, Buffalo, NY USA; 2 Activ Surgical, University at Buffalo, Buffalo, NY USA; 3 Warren Alpert Medical School Alpert Medical School of Brown University, Providence, RI USA; 4 The Ohio State University Wexner Medical Center, Columbus, OH USA

**Keywords:** Health care, Translational research, Medical research

## Abstract

Surgical artificial intelligence (AI) has the potential to improve patient safety and clinical outcomes. To date, training such AI models to identify tissue anatomy requires annotations by expensive and rate-limiting surgical domain experts. Herein, we demonstrate and validate a methodology to obtain high quality surgical tissue annotations through crowdsourcing of non-experts, and real-time deployment of multimodal surgical anatomy AI model in colorectal surgery.

Surgical artificial intelligence (AI) is a nascent field with potential to improve patient safety and clinical outcomes. Current surgical AI models can identify surgical phases, critical events, and surgical anatomy^1–3^. Most of these models utilize supervised machine learning and require large amounts of annotated video data, typically by domain experts. Crowdsourcing, using layperson annotations to form consensus annotations, can scale and accelerate acquisition of high-quality training data^4^.

Crowdsourced annotations of surgical video, however, have historically relied on unsophisticated crowdsourcing methodologies and have been limited to annotations of simple rigid surgical instruments and other non-tissue structures. Models trained to segment laparoscopic surgical instruments performed equally well when trained on non-expert crowdsourced annotations as when trained on expert annotations^5^. However, annotations of deformable and mobile surgical tissues are believed to require domain expertise due to complexity and need for accurate contextual knowledge of surgical anatomy^4^. The acquisition of expert-annotated training data is cost-prohibitive, time consuming, and slows the development and deployment of surgical AI models for clinical benefit.

Here we describe an application of gamified, continuous-performance-monitored crowdsourcing to obtain annotated training data of surgical tissues used to train a soft tissue segmentation AI model. We validate this by training and deploying a highly accurate, real-time AI-assisted multimodal imaging platform to increase precision when assessing tissue perfusion which may help reduce complications such as anastomotic leak in bowel surgery^6,7^.

All video data, composed of 95 de-identified colorectal procedures for benign and malignant indications (IRB #OSU2021H0218), were included for model training (train dataset) and testing (test dataset) (Supplementary Table 1, Methods). Crowdsourced annotations of the train and test dataset were obtained using a gamified crowdsourcing platform utilizing continuous performance monitoring and performance-based incentivization (Fig. 1a, Methods)^8^. Five crowdsourcing parameters were controlled: testing score (TS), running score (RS), minimum crowdsource annotations (n), majority vote (MV), and review threshold (RT) (Fig. 1b, Methods).

**Fig. 1:**
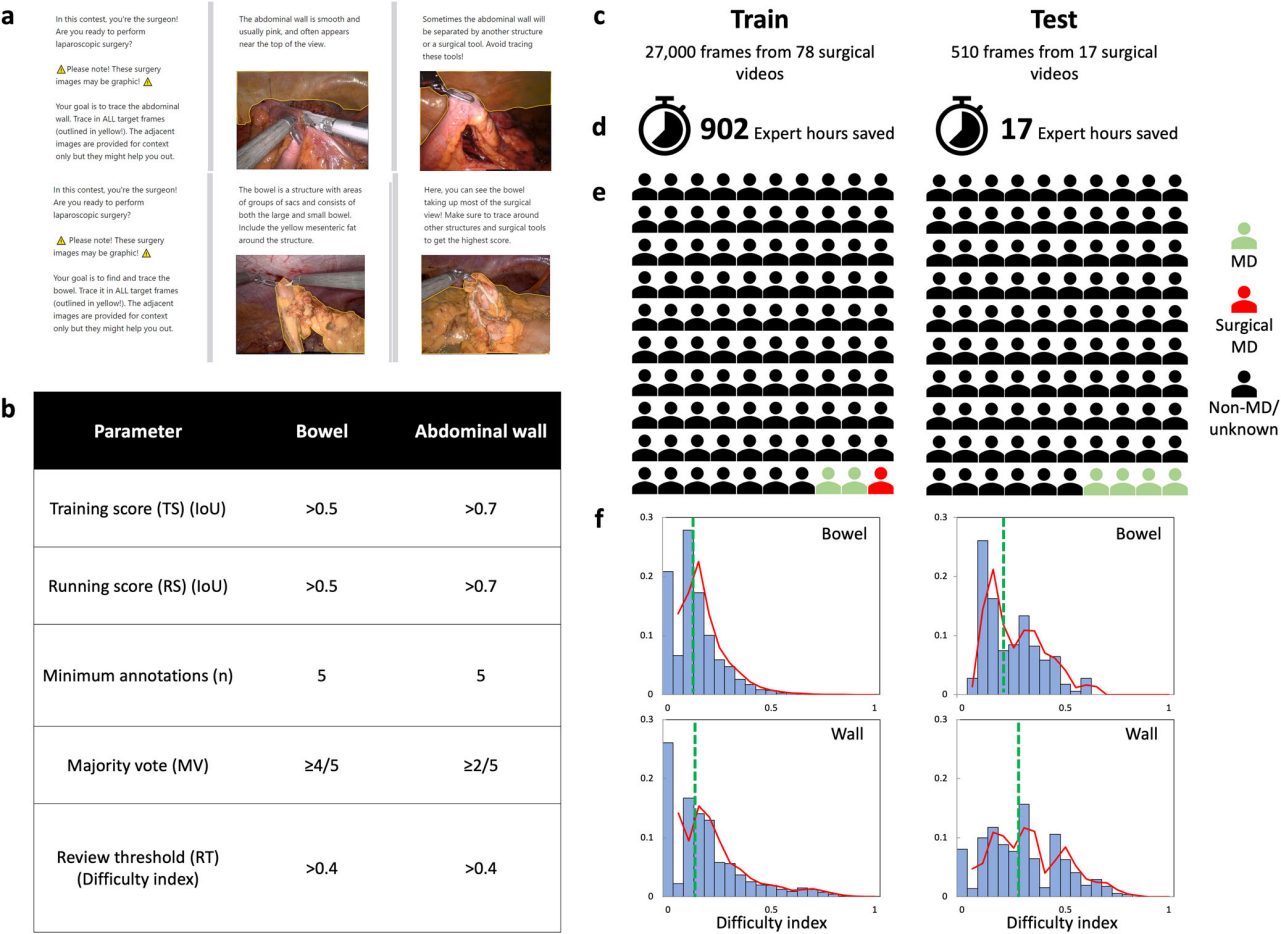
Gamified crowdsourcing methodology and expert time savings. **a** Screenshot images of annotation instructions (Centaur Labs, Boston MA) for bowel and abdominal wall. **b** Crowdsource annotation parameters values used for bowel and abdominal wall tasks. For test and train datasets: **c** Number of videos and frames. **d** Estimated expert hours saved by utilizing crowdsourcing. **e** Crowdsource worker demographics indicating percentage of non-MD/unknown (black), MD (green), and surgical MD (red). **f** Difficulty level (difficulty index) of bowel and abdominal wall (wall) annotations with median values (green dashed line).

Due to the impracticality of time constraints by experts to annotate the large train dataset (27,000 frames), a smaller test dataset (510 frames) was created. This dataset was annotated by crowdsourced workers, the models trained on crowdsourced worker annotations, and one of four surgical experts with surgical domain expertise (Methods). The test dataset was then used to compare the annotations from crowdsourced workers and the models trained from crowdsourced workers to expert annotations. These comparisons were done using standardized metrics of Intersection over Union (IoU) (Supplementary Fig. 1) and the harmonic mean of precision and recall (F1) (Methods, Supplementary Eq. (1)).

*Bowel.CSS* (bowel crowdsourced segmentation), was trained to segment bowel and abdominal wall using crowdsourced annotations of the train dataset. Additionally, a streamlined model was optimized for real-time segmentation of bowel and deployed as a part of an AI-assisted multimodal imaging platform (Methods).

We validate the use of non-expert crowdsourcing with the following primary endpoints:

1. Expertise level of crowdsource workers.
2. Expert hours saved.
3. Accuracy of the crowdsource annotations to expert annotations.
4. Accuracy of the *Bowel.CSS* model predictions to expert annotations.

Secondary endpoints were:

1. Difficulty level of the crowdsourced annotations in the train and test datasets.
2. Accuracy of real-time predictions of the deployed *Bowel.CSS* model to expert annotations.

Train dataset was annotated by 206 crowdsourced workers (CSW) giving 250,000 individual annotations and 54,000 consensus annotations of bowel and abdominal wall. 3% (7/206) of CSW identified as MDs, and 1% (2/206) identified as surgical MDs. Test dataset was annotated by 48 CSW giving 5100 individual annotations and 1020 consensus annotations. 4% (2/48) of CSW identified as MDs, and 0% as surgical MDs (Fig. 1c, e, Supplementary Table 1, Methods).

These demographics indicate non-domain expertise of the CSW. Although demographic data is self-reported and not available for every CSW, the platform reports that the majority of the active CSW are health science students (59.7%) looking to improve their clinical skills (57.3%) (Supplementary Table 2).

On average, an expert spent 120.3 s annotating a frame for bowel and abdominal wall in the test dataset. This extrapolates to an estimated 902 expert hours saved during the annotation of the train dataset by utilizing crowdsourcing methodology, and an estimated 17 expert hours saved in the test dataset (if expert annotations of the test dataset weren’t required for this study). Assuming each of the four expert annotators annotated one hour per day, this estimates to 120 frames annotated per day. In contrast, CSW annotated an average of 774 frames per day in the train dataset (Fig. 1d, Methods).

The difficulty of crowdsourced annotations was measured by Difficulty Index (DI) (Methods). The median difficulty of the crowdsourced annotations was 0.09 DI for bowel and 0.12 DI for abdominal wall in the train dataset, and 0.18 DI for bowel and 0.26 DI for abdominal wall in the test dataset, indicating a robust spectrum of task difficulty across the frame populations. (Fig. 1f, Methods).

Compared to expert annotations of bowel and abdominal wall within the test dataset, crowdsource workers and *Bowel.CSS* were highly accurate; F1 values of 0.86 ± 0.20 for bowel and 0.79 ± 0.26 for abdominal wall for crowdsource workers and 0.89 ± 0.16 and 0.78 ± 0.28 for bowel and abdominal wall for *Bowel.CSS* (Fig. 2a, b).

**Fig. 2:**
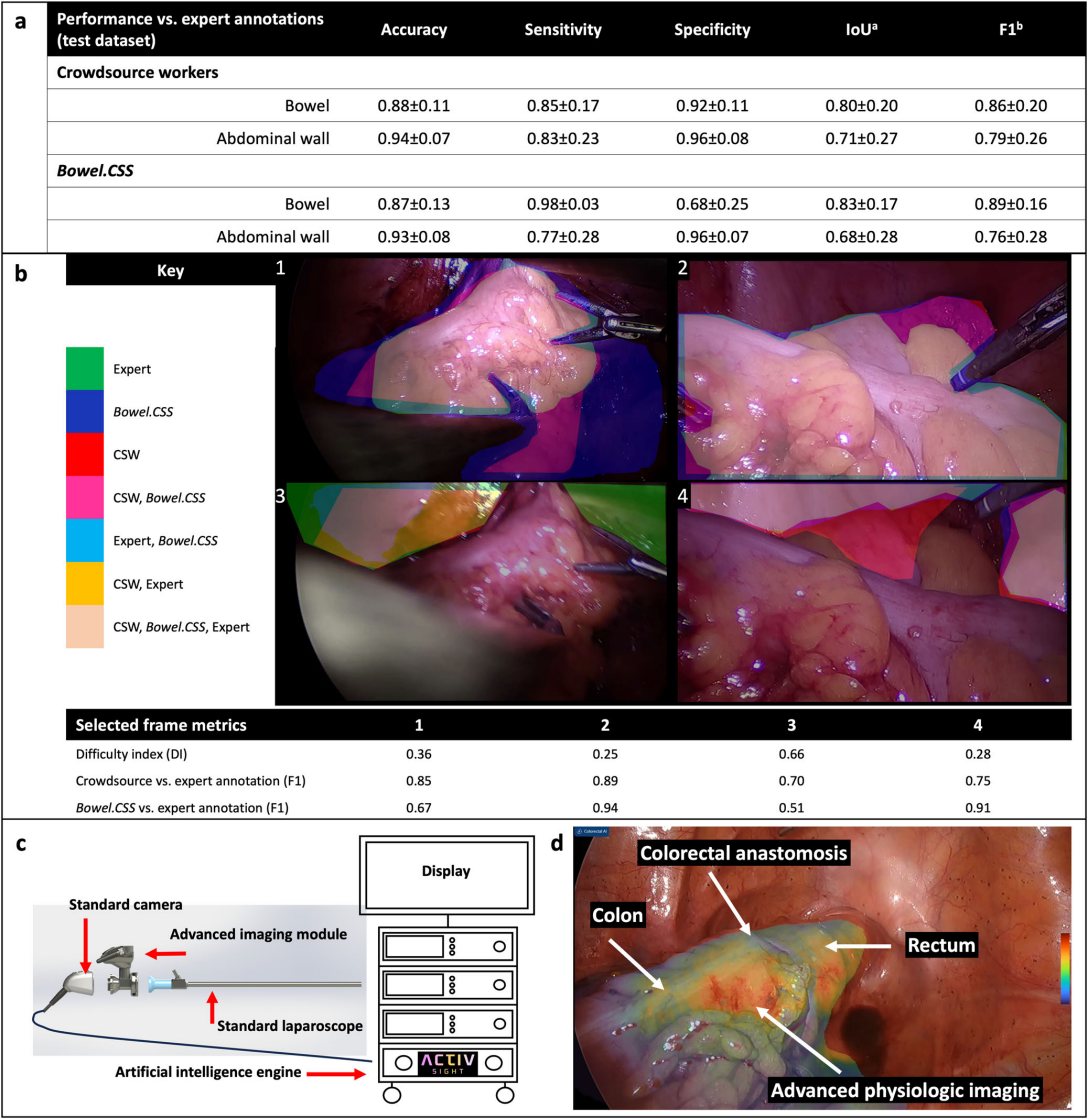
Evaluation of crowdsource and model anatomy segmentations and deployment of near-infrared artificial intelligence system. **a** Crowdsourced annotations and *Bowel.CSS* predictions of bowel and abdominal wall compared to expert annotations in the test dataset. ^a^IoU intersection over union, ^b^F1 dice similarity coefficient. **b** Representative frames comparing crowdsourced annotations and *Bowel.CSS* predictions to expert annotations with corresponding difficulty index. **c** Schematic representing intraoperative deployment of real-time artificial intelligence. **d** Example of deployed version of *Bowel.CSS* incorporated into real-time artificial intelligence assisted multimodal imaging utilizing laser speckle contrast imaging to allow visualization of physiologic information beyond human vision.

A streamlined version of *Bowel.CSS* optimized for real-time bowel segmentation was deployed in real-time to provide AI-assisted display of multimodal imaging and provided highly accurate segmentation of bowel tissue compared to expert annotation. This allowed surgeons to visualize physiologic perfusion the colon and rectum that is normally invisible to human eye (Fig. 2c, d, Supplementary Table 3, Methods).

Herein, we report the first complete and adaptable methodology to obtain highly accurate segmentations of surgical tissues using non-expert crowdsourcing. We outline five crowdsourcing parameters; TS, RS, n, MV, and RT which could be adjusted to fit a variety of segmentations depending on task difficulty and applications. We validated this methodology by showing the crowdsourced annotations can be used to train a highly accurate surgical tissue segmentation model, while greatly accelerating the speed of development by eliminating over 900 expert annotation hours. This study is limited by lack of source video diversity as all videos came from colorectal procedures at a single institution, and thus performance may suffer when applied to other video datasets. Another limitation is the inability to train segmentation models using both crowdsourced and expert annotations due to the inability to source expert annotations for 27,000 video frames in the train dataset due to expert time constraints. However, the crowdsource annotations and the crowdsource trained model predictions were shown highly accurate to expert annotations, and the inability to secure high volume of expert annotations demonstrates the need for crowdsourcing.

While we demonstrated that crowdsourcing is viable when scaling these surgical tissue annotations, further work should be done to determine the limitations of this methodology when applied to increasingly complex anatomical structures. While we showed that the deployed AI model accurately segmented bowel as a part of an AI-assisted multimodal imaging platform, future work should be done to investigate clinical outcomes with the use this technology. This accelerated model development using crowdsource annotations will further enable additional applications of AI-assisted multimodal imaging data for enhanced real-time clinical decision support for safer surgery and improved outcomes.

## Methods

This study was approved by The Ohio State University Institutional Review Board (IRB #OSU2021H0218). All patients provided written informed consent.

### Video source and frame sampling

Surgical videos were obtained from a prospective clinical trial evaluating the utility of real-time laser speckle contrast imaging for perfusion assessment in colorectal surgery (IRB #OSU2021H0218). In the source material for the train dataset, video clips were not prefiltered, and frames were extracted at a regular interval (1 frame per second and 1 frame per 30 seconds) to create a diverse set of training data and eliminate frame selection bias. For the test dataset, clips were extracted when the surgeon was assessing perfusion of the colon. Frames were extracted at 1 frame per second to minimize frame selection bias. The final video and frame counts are represented in Supplementary Table 1.

### Crowdsourced annotations

Crowdsourced annotations of bowel and abdominal wall were obtained using a gamified crowdsourcing platform (Centaur Labs, Boston MA) utilizing continuous performance monitoring and performance-based incentivization^8^. This methodology differs from standard crowdsourcing platforms such as Amazon’s Mechanical Turk, which don’t allow for such continuous performance monitoring and incentivization^9^. Previous implementations of crowdsourcing annotations in surgical computer vision have typically only utilized the majority vote crowdsourcing parameter^5^.

Annotation instructions were developed utilizing as little specialized surgical knowledge as possible while following surgical data science best practices^10^. Crowdsourced annotation instructions given to the crowdsourced workers (CSW) included 13 training steps for each task with 11 and 14 example annotations of abdominal wall and bowel, respectively (Fig. 1a). Four experts (two senior surgical trainees and two trained surgeons) provided expert annotations used to calculate training (TS) and running (RS) scores. In our study, CSW were required to achieve a minimum training score (TS) as measured by intersection-over-union (IoU) with 10 expert annotations prior to performing any annotations. A running score (RS) was calculated by intermittently testing the CSW in the same fashion. Annotations from CSW with a sufficient TS and RS were used in consensus generation. A minimum of 5 annotations (n) were required to generate the consensus crowdsourced annotation using the majority vote parameter (MV) to only include pixels annotated by 4 or more, and 2 or more annotations for bowel and abdominal wall respectively. Difficulty index (DI) was calculated for each frame using IoU with values between 0 and 1, higher indicating increasing difficulty (Supplementary Eq. (2), Methods). Quality assurance (QA) was performed by experts (two surgical trainees) on randomly selected frames above the difficulty review threshold (RT) of 0.4 difficulty index (Fig. 1b).

### SegFormer B3 framework and model training

SegFormer is a semantic segmentation framework developed in partnership with NVIDIA and Caltech. It was selected for the real-time implementation for powerful and yet efficient semantic segmentation capabilities accomplished by unifying transformers with lightweight multilayer perception decoders^11^.

Using the SegFormer B3 framework, we trained two versions of *Bowel.CSS*. *Bowel.CSS* was trained on the entire crowdsource-annotated 27,000 frame dataset (78 surgical videos). A second model, *Bowel.CSS-deployed*, was trained on a subset of the train dataset (3500 frames from 11 surgical videos) and optimized for real-time segmentation of bowel. This model was deployed in real-time as a part of an AI-assisted multimodal imaging platform (Methods).

### Train and test dataset crowdsourced annotations and demographics

Train dataset frames (*n* = 27,000) were annotated by 206 CSW giving 250,000 individual annotations and 54,000 consensus annotations of bowel and abdominal wall. 3% (7/206) of CSW identified as MDs, and 1% (2/206) identified as surgical MDs. Test dataset frames (*n* = 510) were annotated by 48 CSW giving 5100 individual annotations and 1020 consensus annotations. 4% (2/48) of CSW identified as MDs, and 0% as surgical MDs (Fig. 1c, e, Supplementary Table 1, Methods).

To further characterize “unknown” CSW demographics in the crowdsource user population in this study, Supplementary Table 3 presents CSW demographics for the entire annotation platform in the year 2022. It shows the majority (59.7%) were health science students, and the majority listed the reason for participating in crowdsource annotations as “to improve my skills” (57.3%). This supports the conclusion that most users on this platform are non-physicians and are not full-time annotators.

### Crowdsource vs expert hours saved

A primary goal of the use of crowdsourced annotations is to mitigate the rate-limiting and expensive time of experts. The average time for the three domain experts to complete a frame annotation for bowel and abdominal wall was 120.3 s in test dataset. Using the average time to annotate, and the frame totals of 27,000 and 510, crowdsourcing saved an estimated 902 expert hours in the train dataset, and 17 in the test dataset (if experts would have not been required to annotate the test dataset for this study).

### Annotation comparison statistics

The pixel-level agreement of both crowdsourced and *Bowel.CSS* annotations were compared to expert annotation using accuracy, sensitivity, specificity, IoU and F1 scores (Supplementary Fig. 1, Supplementary Eq. (1)). These metrics are accepted measurements of accuracy of segmentation annotations in computer vision and surgical data science^12^.

### Difficulty index

Difficulty of the annotation task was measured per frame using a difficulty index (DI) defined in Supplementary Equation 2 which utilizes the average inter-annotator agreement of the individual CSW annotations to the crowdsourced consensus annotation as measured by IoU. This index is supported by evidence that lower inter-annotator agreement has shown to be an indicator of higher annotation difficulty when other factors such domain expertise, annotation expertise, instructions, platform and source material are constant^13,14^. DI values range from 0 (100% inter-annotator agreement) to 1 (0% inter-annotator agreement). Values closer to 0 indicate easier frames, especially when the annotation target is not visible and the annotation of “no finding” is used since annotations of “no finding” are in 100% agreement. Values closer to 1 indicate harder frames where there is less agreement amongst the CSWs.

The DI of bowel was 0.09 and 0.12 for abdominal wall in the train dataset and was lower than the DI of 0.18 for bowel and 0.12 for abdominal wall in the test dataset. The train dataset included full surgical videos versus the test dataset, which included only clips of surgeons assessing perfusion of the bowel, leading to an increased proportion of “no finding” annotation of bowel (22%) and abdominal wall (32%) in train dataset versus 2.4% and 11% for bowel and abdominal wall in the test dataset. The “no finding” annotations have low difficulty indices leading to the lower median difficulty of the train dataset.

### Real-time deployment of near infrared artificial intelligence

Advanced near infrared physiologic imaging like indocyanine green fluorescence angiography and laser speckle contrast imaging show levels of tissue perfusion beyond what is visible in standard white light imaging. These technologies are used in colorectal resections to ensure adequate perfusion of the colon and rectum during reconstruction to reduce complications and improve patient outcomes. Subjectively interpreting physiologic imaging can be challenging and is dependent on user experience.

*Bowel.CSS* was developed to mask the physiologic imaging data to only those tissues relevant to the surgeon during colorectal resection and reconstruction to assist with interpretation of the visual signal. The output of this model was the bowel label only and it was deployed in real-time on a modified research unit of a commercially available advanced physiologic imaging platform for laparoscopic, robotic, and open surgery.

*Bowel.CSS-deployed* successfully segmented the bowel in real-time during 2 colorectal procedures at 10 frames per second. The intraoperative labels were not saved from the procedures, so to evaluate the intraoperative performance of the model, 10 s clips from each procedure were sampled at 1 FPS (20 frames total) from when the surgeon activated the intraoperative AI model. To assess for accuracy, the model outputs of *Bowel.CSS* and *Bowel.CSS-deployed* were compared to annotations by one of three surgical experts (1 trainee and 2 board-certified surgeons). Model outputs were compared to the expert annotations in these 20 frames using standard computer vision metrics. (Supplementary Table 3).

### Reporting summary

Further information on research design is available in the Nature Research Reporting Summary linked to this article.

### Supplementary information

Supplemental materials

Reporting Summary

## Data Availability

Requests for additional study data will be evaluated by the corresponding author upon request.

## Code availability

The trained Bowel.CSS models are available free and open source (https://github.com/ACTIV-Sugical/Bowel.CSS).
