# Supplementary material for: Real-time near infrared artificial intelligence using scalable non-expert crowdsourcing in colorectal surgery
Source: NPJ Digit Med. 2024 Apr 22;7:99. doi: 10.1038/s41746-024-01095-8 (PMC11035672; doi:10.1038/s41746-024-01095-8)
Supplement: Supplementary file 1 — Supplemental materials [file 41746_2024_1095_MOESM1_ESM.pdf]

# Supplementary tables and figures

## Supplementary table 1

Source videos, annotation and crowdsource worker demographics for train and test datasets.

| Dataset metrics | Train | Test |
|---|---|---|
| **Source videos** | | |
| Frames | 27,000 | 510 |
| Videos | 78 | 17 |
| Procedures | Minimally invasive colorectal procedures | Minimally invasive colorectal procedures |
| **Crowdsource worker demographics** | | |
| Number of annotators | 206 | 48 |
| MD | 3.4% (7) | 4.0% (2) |
| Non-MD[a] | 65.5% (135) | 40.0% (19) |
| Unknown | 31.1% (64) | 56.0% (27) |
| **Annotation demographics** | | |
| Total crowdsource consensus annotations | 54,000 | 1,020 |
| Consensus annotations containing MD annotation | 2.8% (1,527) | 8.0% (82) |
| Consensus annotations without MD annotation | 97.2% (52,473) | 92.0% (938) |
| | | |
| Total crowdsource annotations | 270,000 | 5,100 |
| MD annotations | 0.6% (1,541) | 1.6% (82) |
| Non-MD annotations | 99.4% (268,459) | 98.4% (5,018) |

a = Health science students and non-physician health professionals

## Supplementary table 2

Demographics of crowdsource workers with >100 annotations on the DiagnosUs platform (www.centaurlabs.com) in the year 2022.

| Demographics | Crowdsource workers (n = 838) |
|---|---|
| **Training level** | |
| Health science student | 59.7% (500) |
| Other | 19.9% (167) |
| MD/DO | 12.5% (105) |
| Other health professional[a] | 4.8% (40) |
| No response | 3.1% (26) |
| **Reason for Annotating** | |
| “Improve my skills” | 57.3% (480) |
| “Earn money” | 30.2% (253) |
| No response | 6.9% (58) |
| “Compete with others” | 5.6% (47) |

a = Includes pharmacists, nurse practitioners, and physician assistants.

## Supplementary table 3

Comparison of *Bowel.CSS-v1* and *v2* predictions to expert annotation of 20 frames (10 second clips sampled at 1 frame per second) from two procedures when *Bowel.CSS-v1* was used intraoperatively.

| Performance vs expert annotations (intraoperative frames) | Accuracy | Sensitivity | Specificity | IOU[a] | F1[b] |
|---|---|---|---|---|---|
| ***Bowel.CSS-v1*** **predictions** | | | | | |
| Bowel | 0.87±0.09 | 0.99±0.01 | 0.71±0.23 | 0.83±0.11 | 0.90±0.07 |
| ***Bowel.CSS-v2*** **predictions** | | | | | |
| Bowel | 0.94±0.05 | 0.99±0.01 | 0.87±0.10 | 0.90±0.07 | 0.95±0.04 |
| Abdominal wall | 0.95±0.04 | 0.88±0.17 | 0.98±0.02 | 0.82±0.16 | 0.89±0.11 |

a = Intersection over union; b= Harmonic mean of precision and recall (F1).

## Supplementary equation 1

F1 is the harmonic mean of precision and recall and can be defined by true positives (TP), false positives (FP) and false negatives (FN).

$$\text{F1} = \frac{2 \times Precision \times Recall}{Precision + Recall} = \frac{TP}{TP + \frac{1}{2}(FP + FN)}$$

## Supplementary equation 2

Calculation of Difficulty Index based on average agreement of each crowdsource annotation (Qi) compared to the consensus annotation (C) as measured by Intersection over Union.

$$Difficulty = 1 - \frac{\sum_{i=1}^{n} \frac{|Q_i \cap C|}{|Q_i \cup C|}}{n}$$

*n* = total number of crowdsource annotations that make up the given consensus annotation

## Supplementary figure 1

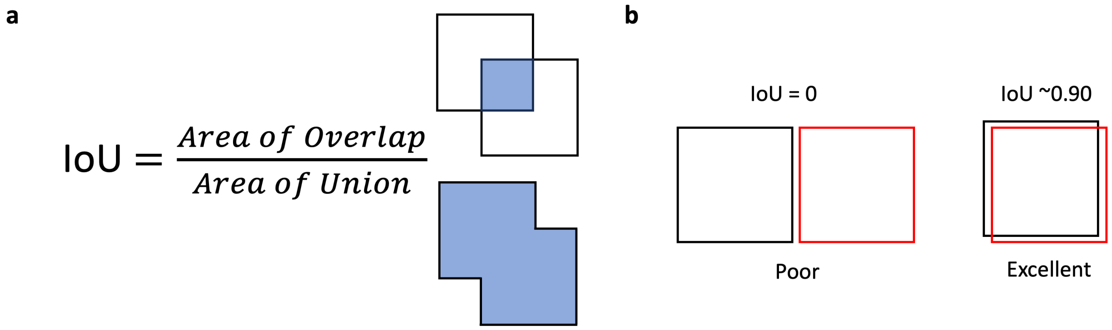


Supplementary Figure 1. A) Intersection over union (IoU) is defined by the area of overlap over the area of union (a). B) IoU ranges from 0 (no overlap) to 1 (perfect overlap).
